# Acquired Hypolipoproteinemia and Hemophagocytic Lymphohistiocytosis: A Case Series and Review

**DOI:** 10.3390/hematolrep17050050

**Published:** 2025-09-22

**Authors:** Leo Reap, Ritwick S. Mynam, Radhika Takiar, Vincent T. Ma

**Affiliations:** 1 Division of Hematology and Oncology, Michigan State University College of Human Medicine, Lansing, MI 49503, USA; lreap1@hfhs.org; 2 Department of Hematology/Oncology, Ascension Providence Hospital, Southfield, MI 48075, USA; 3 Division of Hematology, Medical Oncology, and Palliative Care, Department of Medicine, University of Wisconsin School of Medicine and Public Health, Madison, WI 53726, USA; vtma@medicine.wisc.edu; 4 Division of Hematology and Medical Oncology, Department of Internal Medicine, University of Michigan, Ann Arbor, MI 48109, USA; radhika_takiar@ihacares.com; 5 Department of Dermatology, University of Wisconsin School of Medicine and Public Health, Madison, WI 53726, USA

**Keywords:** case series, hypocholesteremia, HLH, HDL-C, LDL-C, triglycerides

## Abstract

Background: Hemophagocytic lymphohistiocytosis (HLH) is a rare, life-threatening hyperinflammatory syndrome characterized by uncontrolled macrophage activation. Secondary HLH is more common in adults and may be triggered by infection, malignancy, or autoimmune disease. Dyslipidemia, particularly hypolipoproteinemia, has been described but remains underexplored. Methods: We retrospectively reviewed 18 adult HLH cases diagnosed between 2012 and 2020 at two institutions where complete lipid profiles were obtained at or near diagnosis. HLH was defined according to HLH-2004 criteria. Results: Among 18 patients, 17 (94%) had secondary HLH, most commonly idiopathic (*n* = 5, 28%) or Epstein–Barr virus-associated (*n* = 3, 17%). Hypolipidemia was nearly universal: all (18/18) had HDL-C < 30 mg/dL, 15/18 (83%) had HDL-C < 20 mg/dL, and 12/18 (67%) had HDL-C < 10 mg/dL. LDL-C was <100 mg/dL in 12/18 (67%), with 6/18 (33%) undetectable. Triglycerides were variably elevated (median 279 mg/dL, range 96–1658 mg/dL). Three representative cases with profound hypolipoproteinemia demonstrated lipid normalization after HLH-directed therapy. Conclusions: Severe reductions in HDL-C and LDL-C appear to accompany HLH and may contribute to its pathophysiology by impairing antioxidant defenses, destabilizing membranes, and potentiating macrophage activation. This case series highlights a consistent association between hypolipoproteinemia and HLH, suggesting potential diagnostic value. However, the observational design and small cohort limit generalizability. Larger prospective studies are needed to clarify mechanisms and evaluate whether full lipid profiling should be incorporated into diagnostic algorithms.

## 1. Introduction

Hemophagocytic lymphohistiocytosis (HLH) is a rare and life-threatening disorder known to be the result of severe excess macrophage activation [1]. This systemic macrophage activation leads to dysregulated phagocytosis of all different cell lines and the release of numerous pro-inflammatory cytokines, creating a clinical phenotype that mimics an M1 mediated macrophage response and severe sepsis [2].

Broadly, HLH is divided into primary and secondary causes. Primary HLH (pHLH) is associated with genetic defects in perforin or granzyme assimilation and release from natural killer (NK) cells, leading to loss of NK-cell (natural killer cell) mediated control of macrophage activity. pHLH is most common in young children, particularly neonates, but rare cases have been seen as late as 70 years old. Secondary HLH (sHLH) is more common in adults and has numerous causes, including autoimmune disease, rheumatological disorders, cancer, and infections. Furthermore, heterozygous mutations associated with pHLH have been seen in adults with sHLH [3].

However, the definitive pathophysiology of what precipitates hemophagocytosis has not been definitively established [4]. What induces the onset of HLH is unknown, but the precipitating factor often causes significant oxidant stress, commonly acute infection, autoimmune disease, or cancer [5,6,7]. Recently, it was discovered that cytokine-mediated CD-47 (cluster of differentiation 47) downregulation leads to phagocytosis of hematopoietic stem cells (HSC). CD-47, along with signal-protein receptor alpha (SIRPA) has been shown to suppress macrophage-mediated phagocytosis. Severe inflammation downregulates CD-47, potentially leading to phagocytosis of HSC and results in pancytopenia [8]. Furthermore, cholesterol is critical for the association of the three protein components required for the function of CD47 [9]. The membrane spanning the domain of CD-47 binds cholesterol and is required for CD-47 complex formation [10,11]. Without adequate membrane cholesterol, the suppressive function of CD47 may be lost, increasing the rate of cellular phagocytosis [12].

In summary, there appears to be a critical interplay that exists between cholesterol metabolism, inflammation, oxidative stress, and cellular phagocytosis. This interplay may ultimately belie HLH pathophysiology in times of concomitant severe hypolipidemia and oxidative stress.

Herein, we review 18 cases of HLH, and comprehensively highlight 3 subjects, with concomitant severe hypolipoproteinemia and elaborate on the notable association between HLH and hypolipoproteinemia.

## 2. Methods

A retrospective review of cases between 2012 and 2020 was made through the University of Michigan and Ascension Providence Hospital (Michigan) HLH registry, identifying cases of adult HLH where full lipid panels were performed as part of their diagnosis and/or treatment. In total, 18 cases were identified (Table 1) and 3 of those cases are described in further detail below. The diagnosis of HLH was established using the HLH-2004 guidelines [13]. These guidelines require at least 5 out 8 defined clinical and laboratory criteria, which include fever, splenomegaly, cytopenias affecting at least two of three peripheral blood lineages, hypertriglyceridemia and/or hypofibrinogenemia, hemophagocytosis, low or absent NK-cell activity, elevated ferritin, and increased soluble CD25.

## 3. Case Series

### 3.1. Case 1

A 47-year-old man without significant past medical history presented to the hospital with a two-month history of worsening, epigastric pain with distention, fevers, chills, and night sweats. CBC (complete blood count) demonstrated pancytopenia, with hemoglobin of 10.5 g/dL, WBC 2300 cells/μL, ANC 1000 cells/μL, and platelets 45,000 cells/μL. LDH was 770 U/L and haptoglobin was undetectable. Peripheral smear demonstrated true thrombocytopenia and true neutropenia, with marked acanthocytosis with distorted erythrocyte forms and rare schistocytes, <1 per high-powered field. At the time of diagnosis, AST was 289 U/L, ALT 315 U/L, alkaline phosphatase 418 U/L, and ferritin 8330 μg/L. Hepatitis panel was negative.

**Figure 1:**
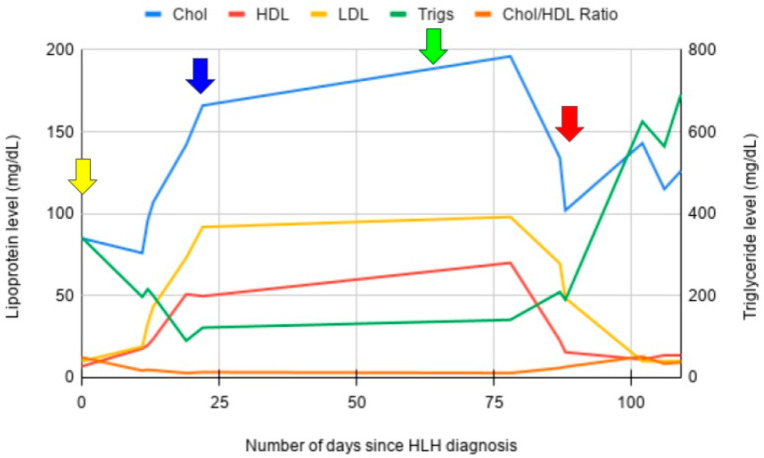
Lipoprotein profile trends for case one during the course of treatment. The yellow arrow denotes time of treatment initiation. The blue arrow denotes time of initial HLH remission. The green arrow denotes time of invasive aspergillosis. The red arrow denotes time of HLH relapse, with subsequent return of near-absent HDL, undetectable LDL, and uptrending triglycerides with disease progression.

The infectious workup was non-revealing. A diagnosis of underlying hemophagocytic lymphohistiocytosis was suspected. A lipid panel was performed to ascertain triglyceride levels. Incidentally, severe hypoalphalipoproteinemia and abetalipoproteinemia were noted, with a total cholesterol of 85 mg/dL, HDL-C (high-density lipoprotein) 6.9 mg/dL, LDL-C (low-density lipoprotein) level undetectable, and triglycerides 342 mg/dL. Trends in his lipid panel were documented over the course of his treatment (Figure 1). Bone marrow biopsy demonstrated a hypercellular marrow with megakaryocytic expansion, decreased M:E ratio, mild dysmorphic features, and clear evidence of hemophagocytosis and phagocytosis of other cell lineages. No clear evidence of malignancy was observed.

CT abdomen/pelvis was negative for hepatosplenomegaly. NK-cell activity returned normal at 9 LU30. Soluble IL-2 receptor was 403 pg/mL (normal <1033 pg/mL). A diagnosis of HLH was confirmed, with five of eight criteria—pancytopenia, fevers, hyperferritinemia, hypertriglyceridemia, and hemophagocytosis. He was started on therapy per the HLH-94 protocol with etoposide and dexamethasone [14]. He made a gradual recovery over the subsequent two weeks and was discharged home. Of note, following treatment with HLH-94 protocol, he had normalization of his lipid profile. He continued on maintenance etoposide and his treatment course was complicated by neutropenic fever with invasive aspergillosis. He was treated with voriconazole and made a full recovery.

He relapsed one month later. Preceding his relapse, HDL-C and LDL-C levels were noted to be downtrending from the baseline. At the time of relapse, his HDL-C fell below 10 mg/dL and LDL-C level became undetectable again. He was started on etoposide and dexamethasone again but had progressive disease with uptrending ferritin. Arrangements were made for him to receive a haploidentical bone marrow transplant. Repeat bone marrow biopsy demonstrated the presence of an occult T-cell lymphoma. Unfortunately, prior to transplantation or subsequent therapy, he developed intracerebral hemorrhage and expired.

### 3.2. Case 2

A 34-year-old woman with a past medical history of hidradenitis suppurativa presented to the hospital with one week of fevers, nausea, vomiting, diarrhea, and a worsening left lower extremity sore. On the day of admission, she developed septic shock, diffuse anasarca, and multiorgan failure, ultimately requiring intubation and vasopressor support.

Ultrasound of her abdomen demonstrated a dilated common bile duct with biliary sludge. ERCP (endoscopic retrograde cholangiopacreatography) was performed and a plastic stent was placed into the ventral pancreatic duct. Cytology was negative for malignancy. She was started on CRRT (continuous renal replacement therapy) with gradual improvement in her renal failure, ultimately thought to be secondary to intravascular depletion accompanied by hypotension and acute tubular necrosis. CT abdomen/pelvis demonstrated splenomegaly, measuring up to 15.9 cm in AP diameter, without mass lesion and mild mesenteric lymphadenopathy.

CBC on admission demonstrated a WBC count of 8150 cells/μL, hemoglobin 11.9 g/dL, MCV 69.8 fL, and platelets 133,000 cells/μL. Differential demonstrated 64% neutrophils, 26% bands, 2% lymphocytes, and 6% monocytes. Peripheral smear demonstrated microcytic, hypochromic anemia with marked acanthocytosis and rare schistocytes, <1 per high-powered field. True mild thrombocytopenia was noted. No blasts were observed. Ferritin was 1332 μg/L, iron 37 mcg/dL, TIBC 73 mcg/dL, and iron saturation 51%. AST was 45 U/L, ALT 48 U/L, alkaline phosphatase 132 U/L, total bilirubin 12.3 mg/dL, and direct bilirubin 9.4 mg/dL. Acute hepatitis panel was negative. Haptoglobin was normal at 192 mg/dL, D-dimer 4637 ng/mL, and fibrinogen 653 mg/dL. Lipid profile revealed severe hypoalphalipoproteinemia and abetalipoproteinemia, with a total cholesterol of 162 mg/dL, HDL-C 7.2 mg/dL, LDL-C level undetectable, and triglycerides 1658 mg/dL.

Following admission, she developed bilateral bullous lesions with associated ecchymoses on her bilateral thighs and sacrum, measuring up to 10 cm in diameter. Infectious and autoimmune workup returned negative. Punch biopsy was obtained, demonstrating prominent epidermal necrosis, superficial dermal necrosis, and necrosis of eccrine coils with prominent dermal hemorrhage and diffuse interstitial neutrophilic infiltrate, felt to be most consistent with acute vasculitis. The definitive etiology of her vasculitis was never identified.

Bone marrow biopsy demonstrated left shifted neutrophilia with lymphocytosis and monocytosis, normocytic anemia, and thrombocytopenia. Mild dysmorphic features were seen with decreased iron stores. No clear evidence of hemophagocytosis was observed. Flow cytometry demonstrated no evidence of acute leukemia or lymphoma. Lumbar puncture cytology was negative for malignant cells.

**Figure 2:**
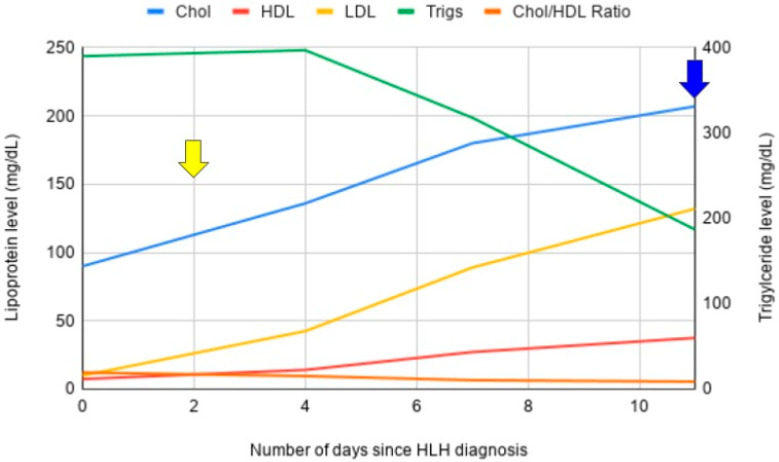
Lipoprotein profile trends for case two. Following treatment initiation, there was progressive normalization of the serum lipoprotein profile. The yellow arrow denotes time of treatment initiation. The blue arrow denotes time of initial HLH remission.

Soluble IL-2 receptor returned at 12,400 pg/mL (normal <1033 pg/mL). Though NK-cell activity was sent, the result was not obtained. Ultimately, a diagnosis of HLH was established with five of eight criteria—fevers, pancytopenia, splenomegaly, hypertriglyceridemia, and elevated soluble IL-2 receptor. She was started on HLH–94 protocol with etoposide and dexamethasone. Following initiation of treatment, she made rapid improvement in her organ function and was able to be extubated. She made a gradual recovery and was able to be discharged home and has continued on maintenance etoposide. Again, of note, following treatment initiation, she had complete normalization of her lipid profile. Trends in his lipid panel were documented over the course of his treatment (Figure 2).

### 3.3. Case 3

A 56-year-old woman with a past medical history of hypertension and diabetes presented with a three-week history of 30-pound unintentional weight loss, intermittent fevers, fatigue, and sore throat. Progressively worsening pancytopenia was noted over the preceding five months. CBC in April 2019 was without abnormality. In January 2020, mild leukopenia was noted with a WBC count of 3370 cells/μL, new onset anemia with a hemoglobin of 11.7 g/dL, MCV 83.2 fL, and platelets 325,000 cells/μL. In May 2020, CBC demonstrated WBC count of 2620 cells/μL, hemoglobin 8.5 g/dL, MCV 75.8 fL, and platelets 255,000 cells/μL. Differential demonstrated 47% PMNs, 2% bands, 44% lymphocytes, and 4% monocytes. Lipid panel obtained in April 2019 demonstrated a total cholesterol level of 143 mg/dL, HDL-C 45 mg/dL, LDL-C 85 mg/dL, and triglycerides 64 mg/dL. Lipid panel obtained in January 2020 demonstrated a total cholesterol of 128 mg/dL, HDL-C 30 mg/dL, LDL-C 75 mg/dL, and triglycerides 117 mg/dL.

At the time of admission, WBC count was 1700 cells/μL, hemoglobin 8.6 g/dL, MCV 78.2 fL, and platelets 148,000 cells/μL. Though previously normal, ANC was 1340 cells/μL. Differential demonstrated 37% PMNs, 1% bands, 49% lymphocytes, and 10% monocytes. Reticulocyte count was 20,700 cells/μL, or 0.60%. PT was 14.5 s, INR 1.2, PTT 41.3 s, and D-dimer greater than 5000 ng/mL. Urinalysis demonstrated packed WBCs, 4+ bacteria, large leukocyte esterase, positive nitrites, and 20–50 RBCs (red blood cells). Transaminitis was observed with AST 282 U/L, ALT 106 U/L, and alkaline phosphatase 47 U/L, LDH was 1538 U/L and there was marked hyperferritinemia with a ferritin of 20,294 μg/L. Lipid panel obtained on admission demonstrated cholesterol 90 mg/dL, HDL-C 7.4 mg/dL, LDL-C undetectable, and triglycerides 390 mg/dL.

**Figure 3:**
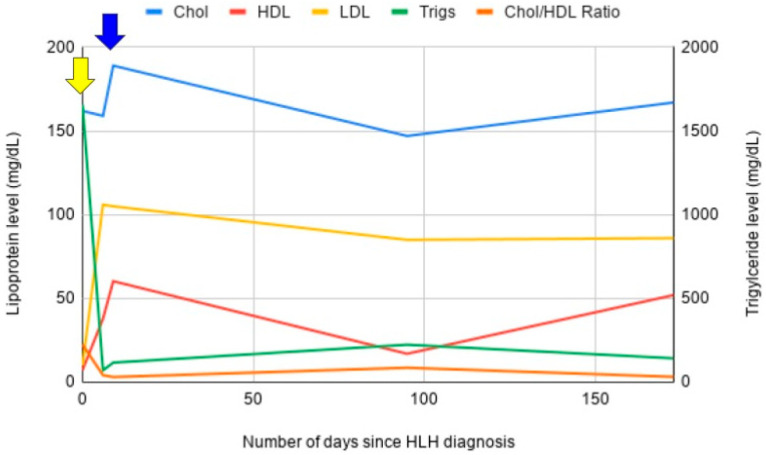
Lipoprotein profile trends for case three. Again, following treatment initiation, there was normalization of the serum lipoprotein profile. The yellow arrow denotes time of treatment initiation. The blue arrow denotes time of initial HLH remission. Lipoprotein profile remained normal thereafter.

A diagnosis of HLH was suspected. A bone marrow biopsy demonstrated a normocytic, normochromic anemia, with increased erythroid maturation. No clear leukemic forms or blasts were observed. Numerous areas of histiocyte consumption of erythroid precursors and other leukocytes were seen, consistent with a diagnosis of HLH. Natural killer cell function was normal. Soluble IL-2 receptor was elevated at 1438 pg/mL. A diagnosis of HLH was established with six out of eight criteria—fevers, pancytopenia, hypertriglyceridemia, hyperferritinemia, hemophagocytosis, and elevated IL-2 receptor. The patient was started on therapy per the HLH-94 protocol with etoposide and dexamethasone. Treatment is still ongoing at present. Following treatment initiation, rapid normalization of the lipid profile was observed. Trends in his lipid panel were documented over the course of his treatment (Figure 3).

## 4. Results

Among the 18 patients in the case series, 17 (94%) had secondary HLH, most commonly idiopathic (*n* = 5, 28%) or Epstein–Barr virus-associated (*n* = 3, 17%). Hypolipidemia was nearly universal. All 18 patients (100%) had HDL-C < 30 mg/dL, 15/18 (83%) had HDL-C < 20 mg/dL, and 12/18 (67%) had HDL-C < 10 mg/dL. LDL-C was <100 mg/dL in 12/18 (67%) and in 6/18 (33%), it was undetectable. Triglycerides were variably elevated, with a median of 279 mg/dL (range: 96–1658 mg/dL).

The three representative cases showed profound hypolipoproteinemia and demonstrated normalization of lipid profiles following HLH-directed therapy. HDL-C and LDL-C levels rose significantly during remission, paralleling clinical improvement. In one patient, relapse was associated with recurrent lipid suppression.

## 5. Discussion

Our case series demonstrates that profound hypolipoproteinemia is a consistent feature of adult HLH, with all 18 patients showing marked reductions in HDL-C and most with severe reductions or complete loss of LDL-C. In the three highlighted cases, lipid profiles normalized with HLH-directed therapy, suggesting these disturbances are dynamic, disease-related, and may parallel disease activity rather than reflecting incidental laboratory abnormalities.

Several biological mechanisms may explain this interplay. During inflammation, circulating LDL-C undergoes oxidative modification, with oxidized LDL-C binding to LOX-1 (lectin-like oxidized LDL-C receptor) on macrophages and driving endocytosis, M1 polarization, and pro-inflammatory cytokine release [15,16]. HDL-C normally mitigates this process through paraoxonase-mediated antioxidant activity [17], but paraoxonase is inhibited in inflammatory states, blunting HDL-C’s protective function [18]. More recent studies have confirmed that oxidized lipid accumulation and impaired HDL antioxidant activity are central mediators of inflammation-related lipid pathology [19]. Identical lipoprotein changes—low HDL-C and LDL-C with variable hypertriglyceridemia—are observed in sepsis [20], systemic lupus erythematosus and rheumatoid arthritis [21], psoriasis [22], and malignancy [23,24], underscoring the generalizability of this inflammatory–lipid axis.

Cholesterol depletion itself has functional consequences beyond altered macrophage signaling. Hypocholesterolemia has been variably defined as a total cholesterol < 120–150 mg/dL or LDL-C < 50 mg/dL [25]. Large population-based cohorts have shown that lipid values vary by age, highlighting the need for context in interpretation [26]. In hematologic conditions characterized by high cellular turnover, such as sickle cell disease [27], β-thalassemia [28], and myelodysplastic syndromes [29], hypolipidemia is thought to reflect increased marrow cholesterol consumption. Similar physiology is observed in the fetus and neonate, where tissue replication creates heightened cholesterol demands and plasma cholesterol is reduced compared to adults [30]. Experimental work has shown that severe cholesterol deficiency increases erythrocyte osmotic fragility and predisposes to hemolysis, whereas lipid repletion normalizes cell membrane stability [31,32]. These phenomena may help explain the cytopenias and membrane fragility seen in HLH.

Cytokine signaling likely provides the unifying framework. Elevated IL-10 (interleukin 10) has been shown to critically regulate circulating lipoproteins, leading to reductions in both LDL-C and HDL-C [33]. While IL-10 is typically anti-inflammatory, IFN-γ (interferon gamma) priming of macrophages can convert its signaling mechanism from STAT3 (signal transducer and activator of transcription 3) to STAT1 thereby activating pro-inflammatory pathways [34]. This may explain why IL-10, normally immunosuppressive, amplifies HLH’s hyperinflammatory state. Both IFN-γ and IL-10 polymorphisms have been linked to HLH susceptibility [35]. In addition, IL-10 directly suppresses NK-cell activity [36], which removes a critical negative feedback mechanism on macrophage activation [37]. Simultaneously, IFN-γ and TNF-α (tumor necrosis factor alpha) inhibit lipoprotein lipase, contributing to hypertriglyceridemia [38]. Deficiency of lipoprotein lipase impairs bone marrow myelopoiesis, potentially contributing to the cytopenias seen in HLH [39]. Together, these cytokine–lipid interactions converge on a model of lipid depletion, impaired antioxidant buffering, loss of CD47–SIRPα inhibitory signaling, and unchecked macrophage phagocytic activity.

Our observations echo the earlier work on primary HLH, where reversible lipoprotein abnormalities were described, with cholesterol enrichment of VLDL-C (very-low density lipoprotein) and severe HDL-C depletion, both normalizing with treatment [40,41]. This mirrors our three representative cases, where lipid abnormalities improved during remission and worsened with relapse.

More recently, independent datasets have extended these findings to clinical outcomes. In adults with secondary HLH, pretreatment HDL-C levels were strongly prognostic, with higher HDL-C associated with improved overall survival [42]. In children, hypocholesterolemia has also been shown to carry prognostic weight: in a cohort of 353 patients, low total cholesterol at diagnosis was an independent predictor of 30-day mortality [43]. In another pediatric study, the combined presence of hypertriglyceridemia and hypofibrinogenemia stratified the risk of multiple organ dysfunction and early death [40]. These findings collectively demonstrate that lipid perturbations are not only diagnostic hallmarks but also prognostic biomarkers across age groups.

Finally, outside HLH, a systematic review of critical illness demonstrated that low total cholesterol, HDL-C, and LDL-C at ICU admission were consistently associated with higher mortality, reinforcing the vulnerability of lipoprotein defenses in systemic inflammation [44].

Thus, lipid derangements in HLH appear both mechanistically plausible and clinically meaningful. Our findings, combined with prior pediatric [39,41,42,43] and adult [40] studies, suggest that hypolipoproteinemia is more than a bystander phenomenon. The consistent and reversible lipid signature across patients, combined with mechanistic plausibility, points toward lipid metabolism as both a diagnostic marker and a potential contributor to HLH pathogenesis.

Despite the significance and potential implications of these findings, the study is limited by its retrospective, observational design and small sample size (*n* = 18). As a result, a causal relationship between hypolipoproteinemia and HLH cannot be established. Furthermore, lipid profiles were not obtained at consistent points in the patients’ disease courses, and severe hypolipidemia also occurs in other inflammatory states, limiting the diagnostic specificity.

## 6. Conclusions

This case series highlights that profound hypolipoproteinemia is a consistent and reversible feature of adult HLH. Reductions in HDL-C and LDL-C were nearly universal in our cohort and normalized with HLH-directed therapy, suggesting a tight link between lipid metabolism and disease activity.

When contextualized with prior pediatric [40,42,43] and adult [41] studies, as well as broader data from critical illnesses [44], these findings strengthen the hypothesis that lipid perturbations are not simply epiphenomena of inflammation but may actively contribute to HLH pathophysiology. Mechanistically, cytokine-driven inhibition of lipoprotein lipase, IL-10-mediated suppression of lipoproteins, oxidative LDL-C uptake, and loss of HDL antioxidant function converge to promote macrophage activation and hemophagocytosis.

Clinically, these findings suggest that lipid profiling could provide a rapid, inexpensive adjunctive tool in HLH diagnosis and possibly in risk stratification; however, specificity is limited, and prospective validation is needed. Future studies should examine whether incorporating full lipid panels into HLH diagnostic criteria improves sensitivity or specificity, and whether thresholds of HDL-C, LDL-C, or total cholesterol carry prognostic value across diverse patient populations.

## Figures and Tables

**Table 1:** Eighteen identified cases of HLH with full lipoprotein profiles performed within proximity to the time of diagnosis.

ID	Sex	Primary or Secondary HLH	Secondary Cause	LDL (mg/dL)	HDL (mg/dL)	Cholesterol (mg/dL)	TG (mg/dL)	Ferritin (ng/mL)
1	Male	Secondary	MDS	10	13	56	279	3106.5
2	Male	Secondary	Cisplatin	110	5	157	208	40,163
3	Female	Secondary	Still’s disease	33	26	78	96	16,500
4	Male	Secondary	EBV	168	5	209	181	16,500
5	Male	Secondary	Intravascular DLBCL	100	9	162	265	1356.9
6	Male	Secondary	EBV	35	5	74	168	11,873.1
7	Female	Secondary	EBV	64	5	124	275	2370.2
8	Female	Secondary	CRC	132	5	171	172	16,500
9	Male	Secondary	Idiopathic	134	5	206	334	33,000
10	Male	Secondary	Idiopathic	54	8	157	747	16,500
11	Female	Secondary	CMV	1	10	57	363	3977.9
12	Female	Secondary	SLE, autoimmune hepatitis	1	5	82	381	1814.2
13	Male	Primary	N/A	58	24	106	120	5868.4
14	Male	Secondary	Viral	35	20	137	1013	14,800
15 *	Male	Secondary	T-cell lymphoma	10	7	85	342	8330
16 *	Female	Secondary	Idiopathic	10	7	162	1658	1332
17 *	Female	Secondary	Idiopathic	10	7	90	390	20,294
18	Male	Secondary	Idiopathic	658	6	727	440	16,500

Abbreviations: LDL—low-density lipoprotein, HDL—high-density lipoprotein, TG—triglyceride, MDS—myelodysplastic syndrome, EBV—Epstein–Barr virus, DLBCL—diffuse large B-cell lymphoma, CRC—colorectal cancer, CMV—cytomegalovirus, SLE—systemic lupus erythematosus. * Highlighted cases.

## Data Availability

The raw data supporting the conclusions of this article will be made available by the authors on request.

## Abbreviations

HDL-C	High-density lipoprotein
LDL-C	Low-density lipoprotein
VLDL-C	Very-low density lipoprotein
ERCP	Endoscopic retrograde cholangiopancreatography
CRRT	Continuous renal replacement therapy
TG	Triglycerides
RBC	Red blood cell
HLH	Hemophagocytic Lymphohistiocytosis
NK-cell	Natural killer cell
CD-47	Cluster of differentiation 47
IL	Interleukin
IFN-y	Interferon gamma
TNF	Tumor necrosis factor alpha
LOX-1	Lectin-like oxidized LDL-C receptor
STAT	Signal transducer and activator of transcription
